# The mental health of NHS staff during the COVID-19 pandemic: two-wave Scottish cohort study

**DOI:** 10.1192/bjo.2021.1079

**Published:** 2022-01-07

**Authors:** Johannes H. De Kock, Helen Ann Latham, Richard G. Cowden, Breda Cullen, Katia Narzisi, Shaun Jerdan, Sarah-Anne Muñoz, Stephen J. Leslie, Neil McNamara, Adam Boggon, Roger W. Humphry

**Affiliations:** Institute for Health Research and Innovation, University of the Highlands and Islands; and Department of Clinical Psychology, New Craigs Psychiatric Hospital, NHS Highland, UK; Nairn Healthcare Group, NHS Highland, UK; Institute for Quantitative Social Science, Harvard University, USA; Institute of Health & Wellbeing, University of Glasgow, UK; Institute for Health Research and Innovation, University of the Highlands and Islands, UK; Institute for Health Research and Innovation, University of the Highlands and Islands, UK; Institute for Health Research and Innovation, University of the Highlands and Islands, UK; Institute for Health Research and Innovation, University of the Highlands and Islands; and Cardiac Unit, Raigmore Hospital, NHS Highland, UK; Department of Psychiatry, New Craigs Psychiatric Hospital, NHS Highland, UK; University College London Medical School, Royal Free Hospital, UK; Epidemiology Research Unit, Scottish Rural College, UK

**Keywords:** Mental health, staff, National Health Service, COVID-19, risk factors

## Abstract

**Background:**

Health and social care workers (HSCWs) are at risk of experiencing adverse mental health outcomes (e.g. higher levels of anxiety and depression) because of the COVID-19 pandemic. This can have a detrimental effect on quality of care, the national response to the pandemic and its aftermath.

**Aims:**

A longitudinal design provided follow-up evidence on the mental health (changes in prevalence of disease over time) of NHS staff working at a remote health board in Scotland during the COVID-19 pandemic, and investigated the determinants of mental health outcomes over time.

**Method:**

A two-wave longitudinal study was conducted from July to September 2020. Participants self-reported levels of depression (Patient Health Questionnaire-9), anxiety (Generalised Anxiety Disorder-7) and mental well-being (Warwick-Edinburgh Mental Well-being Scale) at baseline and 1.5 months later.

**Results:**

The analytic sample of 169 participants, working in community (43%) and hospital (44%) settings, reported substantial levels of depression and anxiety, and low mental well-being at baseline (depression, 30.8%; anxiety, 20.1%; well-being, 31.9%). Although mental health remained mostly constant over time, the proportion of participants meeting the threshold for anxiety increased to 27.2% at follow-up. Multivariable modelling indicated that working with, and disruption because of, COVID-19 were associated with adverse mental health changes over time.

**Conclusions:**

HSCWs working in a remote area with low COVID-19 prevalence reported substantial levels of anxiety and depression, similar to those working in areas with high COVID-19 prevalence. Efforts to support HSCW mental health must remain a priority, and should minimise the adverse effects of working with, and disruption caused by, the COVID-19 pandemic.

The United Nations has warned of a major global mental health crisis as a result of the COVID-19 pandemic.^1^ Government lockdowns, fear of infection, loss of jobs and financial disruptions mean that the public health crisis has negatively affected people in every walk of life.^2^ Not only has the COVID-19 pandemic created adversity, but it has disrupted the most human of our interactions by changing the way people go about their daily lives.

Much speculation, anecdotal reports and preliminary evidence have been circulated about how the public health crisis has affected healthcare providers who are directly involved in managing COVID-19.^3^ Health and social care workers (HSCWs) already exhibited high levels of pre-existing mental health disorders compared with members of the general public,^4–6^ and evidence from previous infectious disease outbreaks suggests that this group is at increased risk of experiencing worse mental health outcomes during the COVID-19 pandemic.^7,8^ Some research suggests that UK front-line staff are experiencing high levels depression, anxiety, stress, burnout and other forms of psychological distress that have been exacerbated by the COVID-19 crisis.^9,10^ In addition, the mental well-being of healthcare workers is likely to have an effect on the national response to the pandemic. It has been shown that high levels of stress and anxiety among HCWs can decrease staff morale, increase absenteeism and negatively affect quality of care.^6^

Most studies on mental health functioning during the pandemic have focused on distress, but the absence of distress (i.e. no depression or anxiety) does not necessarily equate to healthy psychological functioning. We postulate that measuring indices of negative mental health outcomes among HSCWs is of benefit, but that we would do well to do this alongside a measure of positive mental health. The COVID-19 pandemic emerged at a time when there has been increased interest in applying positive psychology and the concept of mental well-being to healthcare workers.^11^ Mental well-being has been found to be protective not only against physical disease, but also against the negative effects of workplace stress, absenteeism and accompanying lower productivity.^12^ Furthermore, mental well-being has been shown to contribute to greater personal resilience, and there is evidence that it can be nurtured in individuals and systems.^13,14^ For HSCWs, mental well-being can assist individuals and systems to develop in a positive way, despite the very real adversity and stress that they are facing in working through this pandemic.

## The present study

There have been several cross-sectional studies during this pandemic that have studied HCWs and their mental health outcomes.^7^^,^^10^ Most have concentrated exclusively on secondary care hospital staff, and there is minimal evidence looking at social care workers and primary care staff.^15^ This is of particular concern in the UK, where a large proportion of COVID-19 deaths have occurred in care homes. There is also a paucity of longitudinal and positive mental health data on HSCWs, which is important for tracking changes in functioning over time.^3,10^ To address some of these gaps in knowledge, this study leverages longitudinal cohort data to provide an assessment of both negative and positive mental health outcomes over time, in a rural National Health Service (NHS) board, looking not just at hospital-based staff, but also incorporating HSCWs from the community. First, we provide an overview of how HSCWs might be coping with working through the COVID-19 pandemic, by tracking mental health outcomes over time. Second, we investigate the sociodemographic determinants of mental health outcomes. This will serve to indicate if any groups of HSCWs should be targeted more specifically to support their mental well-being.

## Method

All procedures involved in this study complied with the ethical standards of the relevant national and institutional committees. The study was approved by Health Research Authority, National Health System UK (approval number 20/SW/0098). All participants provided online informed consent before starting the online questionnaires. They were informed about the purpose and nature of the study, and they had the right to withdraw their data if they wanted to.

### Participants

We recruited a sample of *N* = 225 adult HSCWs to participate in this study. A total of *n* = 56 participants from time point 1 did not complete the survey at time point 2. In this study, the analytic sample included 169 participants for whom we had time point 1 and time point 2 data. Eligibility criteria were being a UK resident aged 18 years or over and working in NHS Highland as an HSCW during the COVID-19 pandemic.

### Measures

Participants were asked to complete a series of demographic and work-related items (i.e. age, gender, household, qualifications, job role, setting, workload burden, having been previously diagnosed with a psychiatric disorder and to what extent the pandemic had affected their job), followed by the psychological measures (time point 1). These participants were contacted via email to participate a second time (time point 2), which involved completing the same psychological measures.

#### Depression

The Patient Health Questionnaire-9 (PHQ-9^16^) was used to measure depression. The nine items ask participants to consider how bothered they have been over the past 2 weeks for each statement (e.g. ‘feeling tired or having little energy’). The questionnaire score ranges from 0 to 27; each question is given a four-point response (‘Not at all’, 0; ‘Nearly every day’, 3). The questionnaire has demonstrated diagnostic validity.^16^ This measure has been used extensively in the UK^10^ and internationally,^17^ to measure levels of depression in various population settings during the COVID-19 pandemic. The PHQ-9 was interpreted as follows: normal (0–4), mild (5–9), moderate (10–14), moderately severe (15–19) and severe (20–27) depression. The cut-off score for detecting symptoms of clinical depression was 10.

#### Anxiety

The seven-item Generalised Anxiety Disorder-7 (GAD-7)^18^ scale was used to measure anxiety. Similar to the PHQ-9, each item asks the respondent to consider the statement based on how much they have been bothered over a 2-week period (e.g. ‘Feeling nervous anxious or on edge’). Each item is scaled from 0 (‘Not at all’) to 3 (‘Nearly every day’), with a total score range of 0–21. The questionnaire has demonstrated diagnostic validity,^18^ and a number of studies have used the GAD-7 to measure levels of anxiety in various UK and international population settings during the COVID-19 pandemic, including those involving front-line healthcare staff.^10,15^ The GAD-7 was interpreted as follows: normal (0–4), mild (5–10), moderate (11–15) and severe (16–21) anxiety. The cut-off score for detecting symptoms of clinical anxiety was 10.

#### Mental well-being

Mental well-being was measured with the Warwick–Edinburgh Mental Well-being Scale (WEMWBS). The scale consists of 14-items used to measure subjective well-being and psychological functioning. The wording of each item is positive, and aimed at addressing positive aspects of mental health. Responses are completed with a five-point scale from 1 (‘None of the time’) to 5 (‘All of the time’); the total score ranges from 14 to 70. The WEMWBS has been validated for use in the UK,^19^ and has been used internationally^20^ and in the UK^21^ to measure the mental well-being of HSCWs during this pandemic. The WEMWBS was interpreted as follows: the cut-off point of ≤40 is indicative of probable depression, and 41–44 indicates possible depression. Scores of 45–59 represent average mental well-being and scores of ≥60 indicate high mental well-being.

### Procedure

Both ‘clinical’ (e.g. doctors, nurses, allied health professionals) and ‘non-clinical’ (administrative) staff were recruited from NHS Highland. Recruitment was supported by NHS Highland Human Resources, general practice managers, NHS Highland social care managers and heads of departments in primary and secondary care, who included links to our study in emails and newsletters. A secondary level of recruitment was conducted on social media: a page for the study was made on Twitter, Facebook and LinkedIn. Interested individuals were directed to a secure data collection website via a weblink, where they first reviewed the study information and gave electronic consent to participate in the study before they completed an online questionnaire. Participant well-being was aligned with ethical committee recommendations of adding an ‘SOS’ button on every page of the online questionnaire, which included local and national mental health support resources, together with the researchers’ contact details. This study was a part of the Scottish Government's Rapid Research into COVID-19, and time restrictions limited recruitment activities to the funding timeframe.

**Fig. 1:**
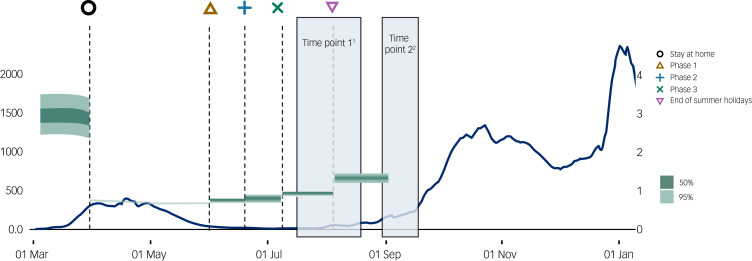
The two measurement periods on the backdrop of infection rates in Scotland. The PHQ-9 (depression), GAD-7 (anxiety) and WEMWBS (mental well-being) was administered at time points 1 and 2. ^1^*N* = 225, *R* = 0.6−0.9, COVID-19 infection growth rate increasing from −0.5 to 0; ^2^*N* = 169, *R* = 0.9−1.5, COVID-19 infection growth rate increasing from −2 to 7. Source: Gov.uk https://coronavirus.data.gov.uk/details/cases?areaType=overview&areaName=United%20Kingdom. GAD-7, Generalised Anxiety Disorder-7; PHQ-9, Patient Health Questionnaire-9; WEMWBS, Warwick–Edinburgh Mental Well-being Scale.

We collected data at two time periods. The first assessment (time point 1) took place from 15 July to 13 August 2020, with most responses (67%) collected between 15 July and 31 July 2020. During time point 1, there was a significant easing of lockdown measures in Scotland (see Fig. 1). During this time, restrictions on outdoor activities were relaxed (28 May) and pubs, restaurants, hairdressers and holiday accommodation were permitted to reopen (10 July).^22^ Time point 2 was approximately 6 weeks later, which occurred from 31 August to 12 September 2020 and coincided with the start of the second wave of the COVID-19 pandemic in Scotland (see Fig. 1), which saw an increase in social restrictions directed by the Scottish Government. During this time, the number of people permitted at indoor and outdoor social gatherings was reduced to six. By 12 September, COVID-19 daily cases in Scotland were at their highest for 4 months.^23^ Local lockdown measures were reintroduced in various Scottish cities (1 September) in response to a rise in cases and hospitality venues.^22^ Fig. 1 provides an overview of the two measurement periods on the backdrop of infection rates and pandemic trends in Scotland.

### Analysis

The outcome variables were the three psychological measures described above, measured at time point 1 and time point 2. The independent (or predictor) variables were the demographic and background variables that were measured only at time point 1. The numbers of individuals were tabulated according to the independent variables, and cross-tabulated between the independent variables where the pairwise combination was deemed of interest.

Our primary interest was in changes in the psychological scores and in providing a parsimonious model for those independent variables that best predicted each of the changes in the dependent psychological variables. There were three stages to this.

Stage 1 involved testing each independent variable in isolation as a predictor of the change in each of the three dependent psychological variables.^24^ First, we looked at *P*-values for univariate models for the changes in each of the three dependent psychological measures (in isolation) with respect to each of the 12 independent variables separately. By looking at the change in the psychological scores, we not only focus on the factor of interest (change), but we also avoid, in a very simple way, the probable difficulty of ‘temporal autocorrelation’. Autocorrelation is the correlation within an individual of their time point 1 score with their time point 2 score. Autocorrelation risks ‘pseudo-replication’, which is when statistical power is erroneously inflated by incorrectly considering correlated replicates as being independent replicates.

Stage 2 involved building a parsimonious model of the change in psychological measures dependent on the independent variables, using the results from stage 1. We used forward stepwise regression to add, in sequence, any independent variable according to their *P*-values (smallest first), among those variables with a *P*-value of <0.05 in their corresponding univariate model from stage 1, and acknowledging that there is an increased risk of a type 1 error, given the number of tests. At this stage we preferred to err on the side of inclusivity. For justifying the retention or otherwise of each of these variables in the multivariable model we used a nested comparison (model with and without the particular independent variable), using an *F*-test with a threshold of the same arbitrary but commonly used *P*-value of 0.05.^25^ Once all of those selected variables had been tested, we then moved onto any remaining variable from the list of those that were considered to be of particular clinical interest^24^ (namely job type, age, working with COVID-19, level of disruption resulting from COVID-19, being female, previous psychiatric disorder, hours of work), and used forward stepwise selection followed by backward stepwise regression to achieve our ‘parsimonious’ model.

Stage 3 is the ‘enriched’ model. In addition to this ‘parsimonious’ model, we wished also to provide an enriched model consisting of the parsimonious model plus all other variables that were considered to be of clinical importance, so that their inclusion was also of interest (despite not being statistically significant). These variables were job type, age, being female, previous psychiatric disorder and hours of work. Missing data within the statistical modelling and the statistical nested comparisons of models was approached as follows: when a relevant independent variable had some values that were missing, those observations that were missing in that variable were removed for both the model with that particular independent variable and for the model without, to ensure that the data on which the model was applied was the same in both cases.

## Results

### Participant demographics

A detailed demographic overview of our sample is provided in Supplementary Appendix 1. Participants were mostly female (88%), over 40 years of age (77%) (Supplementary Table A available at https://doi.org/10.1192/bjo.2021.1079), with a postgraduate degree or higher (62%) and worked mostly as nurses (28%), doctors (23%), allied health professionals (12%), administrative staff (9%), healthcare assistants (5%) and other HSWCs (18%) (Supplementary Table B1). In terms of setting, community, including primary care and general practices (43%), and hospital (44%) accounted for most of the sample (Supplementary Table E2).

### Exploratory association analysis

A detailed exploratory analysis of demographic and professional associations are reported in Supplementary Appendix 2. Of note was that doctors were more likely to be working in primary care or general practices (than in hospitals), whereas nurses were more likely to be working in hospitals (Supplementary Table E2). The majority of the participants were not working directly with COVID-19 (76%), and doctors were more likely to be working with COVID-19 than nurses (Supplementary Table E3). Although the majority of participants worked 30–40 h per week (59%), doctors were the most likely to work more than 40 h per week (Supplementary Table B4). The majority of the sample reported that they were disrupted by COVID-19 either moderately (38%) or majorly (39%), with only 2% reporting no disruption (Supplementary Table C1). Work setting did not appear to be associated with the probability of having been disrupted moderately or majorly by COVID-19 (Supplementary Table C1). In our sample, we found no strong associations with participants reporting having previously been diagnosed with a psychiatric disorder (22% of our sample) and other variables (Supplementary Table B2).

### Prevalence of disease and changes over time (time point 1 to time point 2)

#### Psychological measurements

Table 1 provides a summary of the scores of each psychological measurement over the two time periods, and groups the scores according to severity and clinical (disease) cut-off points.

**Table 1:** Summary statistics for mental health outcomes at both time points

		Mental well-being (WEMWBS)					
	Median (95% CI)^a^	Ordinal classification				Binary classification	
		Probable depression_(<40)_	Possible depression_(41–44)_	Average_(45–49)_	High_(>50)_	Clinical	Non-clinical
Time point 1	45 (43–47)	54 (32%) [25.0–39.6]	29 (17.2%) [11.8–23.7]	76 (45%) [37.3–52.8]	10 (5.9%) [2.9–10.6]	54 (32%) [25.0–39.6]	115 (68%) [60.4–75.0]
Time point 2	44 (42–46)	53 (31.4%) [24.5–38.9]	34 (20.1%) [14.4–27.0]	73 (43.2%) [35.6–51.0]	9 (5.3%) [2.5–9.9]	53 (31.4%) [24.5–38.9]	116 (68.6%) [61.1–75.5]

		Depression (PHQ-9)						
	Median (95% CI)^a^	Ordinal classification					Binary classification	
		Normal_(0–4)_	Mild_(5–9)_	Moderate_(10–14)_	Moderately severe_(15–19)_	Severe_(20–27)_	Non-clinical_(<10)_	Clinical_(>10)_
Time point 1	7 (7–8)	57 (33.7%) [26.6–41.4]	60 (35.5%) [28.3–43.2]	39 (23.1%) [17.0–30.2]	10 (5.9%) [2.9–10.6]	3 (1.8%) [0.4–5.1]	117 (69.2%) [61.7–76.1]	52 (30.8%) [23.9–38.3]
Time point 2	7 (7–8)	54 (32%) [25.0–39.6]	64 (37.9%) [30.5–45.6]	36 (21.3%) [15.4–28.3]	12 (7.1%) [3.7–12.1]	3 (1.8%) [0.4–5.1]	118 (69.8%) [61.5–75.9]	51 (30.2%) [23.7–38.1]

		Anxiety (GAD-7)					
		Ordinal classification				Binary classification	
	Median (95% CI)^a^	Normal_(0–5)_	Mild_(6–10)_	Moderate_(11–15)_	Severe_(16–21)_	Non-clinical _(< 10)_	Clinical _(≥10)_
Time point 1		90 (53.3%) [45.4–61.0]	51 (30.2%) [23.4–37.7]	23 (13.6%) [8.8–19.7]	5 (3%) [1.0–6.8]	135 (79.9%) [73.0–85.6]	34 (20.1%) [14.4–27.0]
Time point 2		79 (46.7%) [39.0–54.6]	53 (31.4%) [24.5–38.9]	27 (16%) [10.8–22.4]	10 (5.9%) [2.9–10.6]	123 (72.8%) [65.4–79.3]	46 (27.2%) [20.7–34.6]

WEMWBS, Warwick–Edinburgh Mental Well-being Scale; PHQ-9, Patient Health Questionnaire-9; GAD-7, Generalised Anxiety Disorder-7.

a. Confidence intervals around the median were calculated by bootstrapping (number of simulations = 1000).

The original scores of the outcomes are presented as medians with confidence intervals in Table 1, and indicate overall group scores on the PHQ-9 in keeping with mild depression (7.0, 9%% CI 7–8) for the first measurement and again (7.0, 95% CI 7–8) for the second measurement. The median scores on the GAD-7 for anxiety were in keeping with high-normal levels of anxiety (5.0, 95% CI 4–6) for the first measurement and mild anxiety (6.0, 95% CI 5–7) for the second measurement. The median scores on the WEMWBS for well-being were in keeping with low-average levels of psychological well-being (45.0, 95% CI 43–47) for the first measurement and indicative of low levels of well-being (44.0, 95% CI 42–46) for the second measurement. Although the aggregated scores of the three psychological measures (depression, anxiety and mental well-being ) indicated little overall change in the group of individuals between the two time periods, we observed severity category changes for anxiety and mental well-being over time.

**Fig. 2:**
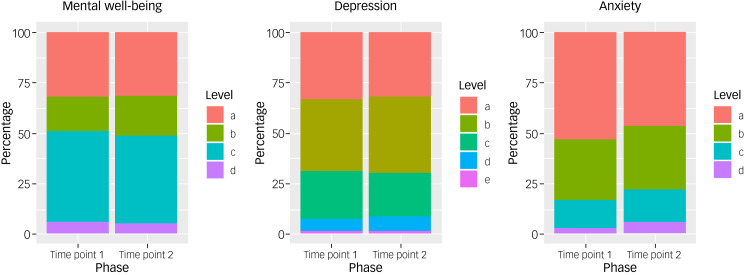
Main results of WEMWBS (mental well-being), PHQ-9 (depression) and GAD-7 (anxiety), and at time points 1 and 2, presented as proportion of participants with scores in different subcategories at different time points. Mental well-being: (a) probable depression (≤40), (b) possible depression (41–44), (c) average mental well-being (45–59), (d) high mental well-being (≥60); depression: (a) normal (≤4), (b) mild depression (5–9), (c) moderate depression (10–14), (d) moderately severe depression (15–19), (e) severe depression (≥20); anxiety: (a) normal anxiety (≤4), (b) mild anxiety (6–10), (c) moderate anxiety (11–15), (d) severe anxiety (≥16). GAD-7, Generalised Anxiety Disorder-7; PHQ-9, Patient Health Questionnaire-9; WEMWBS, Warwick–Edinburgh Mental Well-being Scale.

Figure 2 elucidates Table 1 by presenting the psychological measurements as proportion of participants with scores in different subcategories at different time points. Of our sample, 30.8% reported scores in keeping with probable depression^16^ at time point 1, and it remained constant at 30.2% at time point 2. Of note here is that a further 35.5% of the sample reported symptoms in keeping with mild depression (scores ranging from 5 to 9 on the PHQ-9) at time point 1 and 37.9% at time point 2.

In our sample, 20.1% reported scores in keeping with probable anxiety^18^ at time point 1, increasing to 27.2% at time point 2. Of note here is that 30.2% of the sample reported symptoms in keeping with mild or subclinical anxiety (scores ranging from 6 to 10 on the PHQ-9) at time point 1 and 31.4% at time point 2.

**Fig. 3:**
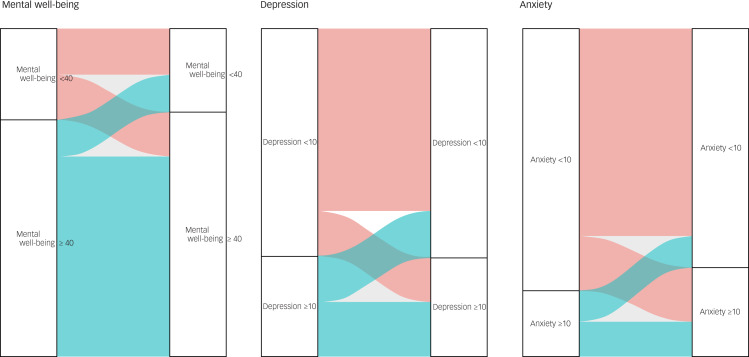
A visual representation of the changes in clinical states for mental well-being (WEMWBS score <40), depression (PHQ-9 score ≥ 10) and anxiety (GAD-7 score ≥ 10), and between time point 1 and time point 2. GAD-7, Generalised Anxiety Disorder-7; PHQ-9, Patient Health Questionnaire-9; WEMWBS, Warwick–Edinburgh Mental Well-being Scale.

On the WEMWBS, our sample reported average (45%) and high (5.9%) mental well-being at time point 1, with a slight decrease in average (43.2%) and high (5.3%) mental well-being at time point 2. At time point 1, 31.9% of participants reported scores in keeping with probable depression (scores of ≤40 on the WEMWBS), and 17.2% of participants reported scores in keeping with possible depression (scores of ≤44) on the WEMWBS. At time point 2, these reported scores remained constant for probable depression (31.4%), with a slight increase in possible depression (20.1%). Figure 3 provides a visual representation of the changes for each psychological measure dichotomised into clinical (disease) and non-clinical states over time. The diagram demonstrates, for example, that about half of the individuals who met the threshold for low mental well-being (scores <40) in time point 1 ‘moved’ to not meeting the disease threshold (≥40) at time point 2, but these were approximately ‘replaced’ by individuals ‘moving’ in the opposite direction, so the overall make-up of the group remained stable. A similar pattern is seen in the depression and anxiety measures. For the interested reader, Supplementary Appendix 2, Table 2 provides the exact number of individuals moving from one state to the other over the duration of our study.

#### Correlations

In our sample, mental well-being was strongly negatively correlated with depression and anxiety (Table 2).

**Table 2:** Correlations (Pearson coefficient) with corresponding 95% confidence intervals for data over each time period, of each psychological measure against one another

	Mental well-being	Depression	Anxiety
Time point 1			
Mental well-being	1		
Depression	−0.71 (−0.78 to −0.63)	1	
Anxiety	−0.61 (−0.70 to −0.51)	0.71 (0.63–0.78)	1
Time point 2			
Mental well-being	1		
Depression	−0.75 (−0.81 to −0.67)	1	
Anxiety	−0.64 (−0.73 to −0.55)	0.73 (0.65–0.79)	

### Risk factors

#### Summary statistics for risk factors

Summary statistics for the main risk factors are provided in Supplementary Tables A–F1 within the appendixes. A pairwise cross-tabulation is presented for combinations of demographic variables that were of prior interest (Supplementary Tables A–F1).^7,15^ Univariate associations between the risk factor (independent variables) and the change in the dependent variables (psychological scores) can also be found here. The results of the 36 univariate models (36 pairs = 12 predictors×3 outcomes) are presented in Supplementary Appendix 2, Table 3. Two out of the 36 pairs had *P*-values of <0.05. These were working with COVID-19 and being disrupted by COVID-19 (see Supplementary Appendix 2, Table 3).

### Accounting for confounding factors

#### Multivariable models

The forward stepwise regression led to the choice of our parsimonious models (Tables 3–6). The reader is reminded that by focusing on the variable of interest, which was the change in psychological score, we effectively accounted for any possible temporal autocorrelation (see Method section). These parsimonious models were enriched with the variables of particular clinical interest, to present our enriched models (Tables 4, 6 and 7)

**Table 3:** Selected parsimonious model of change in mental well-being scores over time

	Estimate	s.e.	95% confidence interval	*P*-value
Intercept	12.3	5.1	(2.28–22.38)	0.02
Disruption resulting from COVID-19				
No disruption from COVID-19	Reference	−		−
Minor disruption	−15.5	5.6	(−26.47 to −4.46)	0.006
Moderate disruption	−11.9	5.2	(−22.20 to −1.64)	0.02
Major disruption	−13.3	5.2	(−23.58 to −3.03)	0.01
Severe disruption	−9.7	5.5	(−20.51 to 1.04)	0.08

**Table 4:** The estimates and confidence intervals for the selected parsimonious model for the change in mental well-being (time point 2 – time point 1), enriched with additional predictor variables of particular clinical interest

	Change in mental well-being	95% confidence interval
(Intercept)	9.96	
Minor disruption resulting from COVID-19	−14.74	(−26.42 to −3.07)
Moderate disruption resulting from COVID-19	−12.12	(−23.14 to −1.11)
Major disruption resulting from COVID-19	−12.92	(−23.84 to −2.01)
Severe disruption resulting from COVID-19	−9.88	(−21.52 to 1.76)
Additional predictor variables of particular clinical interest		
Job: doctor	0.91	(−5.21 to 7.03)
Job: nurse	0.95	(−4.65 to 6.55)
Job: carer	0.16	(−8.6 to 8.92)
Job: healthcare assistant	−0.19	(−8.34 to 7.97)
Job: allied health professional	2.13	(−4.21 to 8.47)
Job: other	1.47	(−4.22 to 7.17)
Job setting: hospital and primary care	−0.43	(−3.73 to 2.88)
Job setting: other	−0.82	(−5.76 to 4.11)
Psychological disorder	−0.51	(−3.91 to 2.89)
Gender	−2.79	(−7.25 to 1.67)
Education level	−0.5	(−3.97 to 2.96)
Hours worked: between 20 and 30	7.25	(0.04–14.46)
Hours worked: between 30 and 40	4.25	(−2.44 to 10.95)
Hours worked: over 40	7.11	(−0.41 to 14.63)

.

#### Mental well-being

The selected multivariable model of change in well-being scores over time is presented in Tables 3 and 4.

Table 4 presents the estimated effect of each variable on the change in anxiety and the corresponding 95% confidence interval for that change.

An ANOVA test of this model with and without the change in the ‘disruption resulting from COVID-19’ variable confirmed that, even with the addition of these additional predictors, the variable ‘disruption resulting from COVID-19’ was statistically significant (*P* = 0.01).

These results suggests that individuals with no disruption from COVID-19 experienced an increase in mental well-being score between the two time periods, whereas other individuals who reported disruption resulting from COVID-19 experienced a decrease in mental well-being.

#### Depression

The selected parsimonious model for depression is presented in Table 5. Table 6 presents the estimated effect of each variable on the change in depression and the corresponding 95% confidence interval for that change. An ANOVA test of this model with and without the change in working with COVID-19 variable confirmed that even with the addition of these additional predictor variables, working directly with COVID-19 was still statistically significant (*P* = 0.008).

**Table 5:** Selected model of change in depression scores over time

	Estimate	s.e.	95% confidence interval	*P*-value
Intercept	−4.0	1.7	(−7.31 to −0.77)	0.01
Work with COVID-19: no	Reference	−		−
Work with COVID-19: yes	2.4	0.9	(0.58–4.17)	0.01

**Table 6:** Enriched model of change in depression over time (parsimonious model enriched with variables of prior interest)

	Change in depression	95% confidence interval
(Intercept)	−10.07	(−19.08 to −1.06)
Working directly with COVID-19	2.8	(0.74–4.87)
Minor disruption resulting from COVID-19	4.95	(−1.5 to 11.41)
Moderate disruption resulting from COVID-19	4.07	(−2.03 to 10.17)
Major disruption resulting from COVID-19	3.69	(−2.36 to 9.74)
Severe disruption resulting from COVID-19	3.83	(−2.63 to 10.3)
Job: doctor	3.54	(0.04–7.04)
Job: nurse	1.37	(−1.75 to 4.48)
Job: carer	0.42	(−4.43 to 5.28)
Job: healthcare assistant	1.82	(−2.77 to 6.4)
Job: allied health professional	0.41	(−3.12 to 3.94)
Job: other	−0.08	(−3.22 to 3.07)
Job setting: hospital and primary care	0.39	(−1.46 to 2.23)
Job setting: other	1.13	(−1.61 to 3.87)
Psychological disorder (yes)	0.46	(−1.44 to 2.36)
Gender	2.32	(−0.15 to 4.78)
Education level	−0.61	(−2.54 to 1.31)
Hours worked: between 20 and 30	−2.32	(−6.34 to 1.69)
Hours worked: between 30 and 40	−2.48	(−6.21 to 1.24)
Hours worked: over 40	−4.26	(−8.44 to −0.08)

#### Anxiety

Table 7 presents the model of change in anxiety over time enriched with all variables of interest. The estimated effect of each variable on the change in anxiety and the corresponding 95% confidence interval for that change.

**Table 7:** Model of change in anxiety over time enriched with all variables of interest (the parsimonious model supported no independent variables)

	Change in anxiety	95% confidence interval
(Intercept)	−4.94	−12.55 to 2.68
Minor disruption resulting from COVID-19	4.84	−1.2 to 10.88
Moderate disruption resulting from COVID-19	3.96	−1.73 to 9.66
Major disruption resulting from COVID-19	4.2	−1.45 to 9.85
Severe disruption resulting from COVID-19	2.81	−3.21 to 8.84
Job: doctor	2.48	−0.68 to 5.65
Job: nurse	1.16	−1.74 to 4.05
Job: carer	2.92	−1.62 to 7.45
Job: healthcare assistant	3.82	−0.4 to 8.03
Job: allied health professional	0.62	−2.66 to 3.9
Job: other	2.44	−0.51 to 5.38
Job setting: hospital and primary care	−0.95	−2.66 to 0.76
Job setting: other	0.51	−2.05 to 3.06
Psychological disorder	−0.4	−2.16 to 1.36
Gender	1.09	−1.22 to 3.4
Education level	0.08	−1.71 to 1.87
Hours worked: between 20 and 30	−0.33	−4.05 to 3.4
Hours worked: between 30 and 40	−0.64	−4.1 to 2.82
Hours worked: over 40	−2.87	−6.76 to 1.02

The overall change in anxiety between time point 1 and time point 2 was not significantly different from zero (*P* = 0.07), and none of the tested independent variables were associated with significant changes in anxiety. These data suggested that anxiety was not statistically significantly influenced by or associated with time or any of the measured variables.

In summary, it appears that being disrupted by COVID-19 was an important factor associated with the size and direction of change (decrease) in mental well-being. Working with COVID-19 was an important factor in change (increase) in depression measures between time point 1 and time point 2.

## Discussion

The aim of this study was to examine the mental health functioning of rural Scottish HSCWs during the COVID-19 pandemic over time. To this end, we tracked changes in mental health outcomes over two time points and explored the determinants of those outcomes in a cohort of HSCWs working in the Scottish Highlands. Other large-scale studies in areas with high COVID-19 infection rates have generally reported an increase in the prevalence of adverse mental health outcomes (i.e. depression, anxiety and psychological distress) in this population during the COVID-19 pandemic.^10^ Evidence from previous epidemics,^7,26^ together with pre-pandemic data, indicate a high and persistent burden of psychological distress among HSCWs,^4,8^ placing them at risk for exacerbated adverse mental health outcomes during this pandemic.^5^ Our findings corroborate the existing literature by reporting substantial levels of probable depression and anxiety in our HSCW cohort over time. Our results add to the existing literature by indicating that HSCWs working in areas outside of COVID-19 hotspots experience levels of adverse mental health outcomes in keeping with those working in COVID-19 hotspots. Previous studies have identified determinants of mental health outcomes,^7,10^ and our results add longitudinal evidence that groups at increased risk of adverse mental health outcomes are those working directly with COVID-19 and those whose work roles have been disrupted by the pandemic.

### Prevalence of disease

Our analytic sample reported relatively high levels of anxiety and depressive symptoms^10^ that persisted over time. Although there was no statistically significant change in participants’ levels of anxiety, depression and mental well-being between two time points in summer and autumn of 2020, we found category changes of clinical interest in our cohort's levels of anxiety (increasing from normal to mild) and mental well-being (decreasing from average to low). Participants meeting the cut-off for probable anxiety increased from 20.1% at time point 1 to 27.2% at time point 2.

Levels of self-reported symptoms in line with probable depression did not change between time points 1 and 2 (prevalence 30.8% and 30.2%, respectively, as reported on the PHQ-9). These findings were corroborated in our cohort's mental well-being scores, where we found that over 30% of HSCW who responded to our survey reported low levels of mental well-being consistent with probable clinical depression over both time periods. This finding suggests that levels of probable depression and mental well-being for HSCWs in the Scottish Highlands during the COVID-19 have been worse than in the general population of Scotland during the pre-pandemic period.^27,28^ Public Health Scotland published a rapid review of the impact of the COVID-19 pandemic on population mental health.^29^ Although the review found little good-quality longitudinal evidence to suggest changes in the prevalence of population-level mental health outcomes caused by the pandemic, it identified health and social care staff as an at-risk group of adverse mental health outcomes. Although direct comparison of the mental health of our cohort and the general Scottish population during the pandemic is currently limited, our results are suggestive that our cohort's levels of depression were broadly in line with those seen internationally for healthcare workers during the pandemic.^17^

Our data also permits comparison with the point-prevalence levels of depression and anxiety among HSCWs during the COVID-19 pandemic nationally and internationally. Luo et al^17^ conducted a systematic review and meta-analysis on the mental health impact of COVID-19 on health workers, the general population and patients at high risk of COVID-19.^17^ They reviewed 62 studies published between 1 November 2019 and 25 May 2020 and included 162 639 participants from 17 countries. The pooled prevalence of anxiety was 33% and the pooled prevalence of depression was 28%. Studies from China, Turkey, Spain, Italy and Iran reported higher than pooled prevalence of anxiety and depression among health workers compared with the general public.^17^ However, this finding has not been replicated across all contexts; in Germany, for example, Skoda et al found that healthcare professionals showed less anxiety and depression than non-healthcare professionals.^27,30^ Respondent data from the Scottish Highland sample suggest levels of depression broadly in line (slightly higher) with the international pooled prevalence and slightly lower than the average for anxiety. This study also adds to current literature, confirming that anxiety and depression symptoms are a concern for the healthcare service in the Scottish Highlands during this pandemic. In Scotland, there has been a steady increase in the proportion of the adult population who report two or more symptoms of depression since the Scottish Health Survey began reporting data on this measure (from 8% in 2010–2011 to 11% in 2016–2017).^27^

This paper also adds, to a limited degree, a longitudinal perspective, demonstrating that our cohort's symptoms remained fairly stable over time and that there is a general trend toward worsening mental health outcomes with regard to anxiety and mental well-being. These findings are of concern, as sustained symptoms of low mental well-being and anxiety are more likely to lead to long-term psychological disorders, and are likely to contribute to absenteeism and low morale in the workplace.^6,8^ Of note is that baseline data was collected during a period of easing of lockdown restrictions, and follow-up was completed at the start of the second wave of infections, when lockdown measures were reintroduced in Scotland. The timing of data collection is likely to have had an effect on mental health rates. Our results, together with previous studies,^31^ suggest that changes in mental health outcomes during the COVID-19 pandemic might depend on the timing of assessments within particular contexts, as well as the population groups assessed. Other UK studies have found that the general population reported worse levels of psychological health during the initial ‘shock’ of the pandemic, followed by consistent trends toward pre-pandemic levels of depression and anxiety.^31^ Although we did not collect data on this sample from before the COVID-19 pandemic, we speculate that front-line staff may find it particularly difficult to return to pre-pandemic levels of psychological health. In contrast to what was found in the UK general population, our study reported sustained substantial levels of depression and anxiety, and low levels of mental well-being over time. The prolonged second wave in the UK, high levels of hospital admissions, persistent social distancing regulations, and the added pressure of providing a nationwide vaccination program together with managing increasing pressure from non-COVID-19 health issues on the health service leads to concerns about worsening mental health for our HSCWs.

### Determinants of mental health outcomes

A further aim for this study was to identify determinants our population's mental health outcomes over time. The two variables that were statistically significant risk factors over time, and when accounting for confounding factors, were disruption caused by COVID-19 and working directly with COVID-19. This appears to confirm concerns that the pandemic itself is contributing to poor mental health among HSCWs.

### Proximity to patients with COVID-19

Working directly with patients with COVID-19 has been found to be a risk factor for poor mental health outcomes in previous studies.^7,15^ This may be exacerbated by a fear of infection (to self and others), which has also been found to be a risk factor in previous studies.^7,15^ Our study corroborated previous research, showing that working directly with COVID-19 was significantly associated with higher rates of depression. These findings could point toward the use of monitoring the mental health and providing additional psychological support for departments that work directly with COVID-19.^7,15^

Although it is of interest that this study confirms direct contact with patients with COVID-19 as a risk factor for poor mental health outcomes in HSCWs, it is important to note that NHS Highland is a region with relatively low numbers and rates of COVID-19 infection, and the majority of our cohort (76%) did not work directly with COVID-19. From the beginning of the COVID-19 pandemic to 12 June 2021, NHS Highland has recorded 5419 cases of COVID-19 in total, at an infection rate of approximately 1614 per 100 000 population (the fifth lowest rate in the UK).^23,32^ In Scotland there have, in the same time period, been 245 744 cases at a rate of 4498 per 100 000 population,^23,32^ and in the UK as a whole there have been 4.54 million cases at a rate of 6824 per 100 000 population.^23,33^ Unlike our cohort, the majority of studies thus far have studied urban, secondary care populations of HCWs in areas of high COVID-19 rates.^15^ In a recent study, Lamb et al examined the mental health in a large sample of healthcare staff working in areas with high COVID-19 rates.^10^ Our study's findings suggest levels of depression and anxiety similar to those reported in COVID-19 hotspots.^10^ This suggests that, although direct contact with patients with COVID-19 is a risk factor, there are likely to be other, indirect factors contributing to adverse mental health outcomes in this cohort outside of being a HSCW, and the likelihood thereof should not be negated in our study's results; for example, at the beginning of time point 1 (15 July) there were 772 new cases of COVID-19 in England, and at the end of time point 1 (13 August) there were 1138 cases. During time point 1 the average number of new COVID-19 cases in England per day was 835.^23,33^ In Scotland, at the beginning of time point 1 (15 July) there were 16 new cases of COVID-19, and at the end of time point 1 there were 52 cases. The average number of daily cases in Scotland during time point 1 was 28 (total: 910).^32^ In NHS Scotland, the average number of daily hospital admissions attributed to COVID-19 during time point 1 was 3.0. In NHS Highland at the beginning of time point 1 there were zero new cases of COVID-19 recorded, and at the end of time point 1 there were two new cases. The average number of daily cases in NHS Highland during time point 1 was 0.5 (total: 15).^32^ At the beginning of time point 2 (31 August) there were 1501 new cases of COVID-19 in England, and at the end of time point 2 (12 September) there were 2656 cases. During time point 2 the average number of new COVID-19 cases in England per day was 2912.^23^

In Scotland, at the beginning of time point 2 (31 August) there were 144 new cases of COVID-19, and at the end of time point 2 (12 September) there were 207 new cases. The average number of daily cases in Scotland during time point 2 was 178 (total: 2319).^32^ In NHS Highland, at the beginning of time point 2 there were seven new cases of COVID-19 recorded, and at the end of time point 2 there were 15 new cases. The average number of daily cases in NHS Highland during time point 2 was 6.6 (total: 86).^32^ Across NHS Scotland, the average number of daily hospital admissions attributed to COVID-19 during time point 2 was 10.^23,32^

Although there was an increase in COVID-19 incidence and hospital admission between time point 1 and time point 2 in the UK as a whole, in Scotland and in NHS Highland, the Scottish Highlands were not directly affected to a significant degree by clinical cases of COVID-19 during either period addressed in this study. NHS Highland serves a large but widely dispersed population, most of whom live far from the major metropolitan areas most affected by the pandemic. It may be that this relative degree of isolation represented a protective factor, although our paper was not designed to investigate this hypothesis. Given the relatively low local incidence and prevalence of COVID-19 during the study period, it may be that the increased incidence in poor mental health observed was also a result of the general societal impact of the pandemic.

### Disruption resulting from COVID-19

Self-reported subjective levels of disruption may go some way toward explaining the high levels of depression, increases in anxiety and decreases in mental well-being in our cohort. Our findings show that disruption resulting from COVID-19 was significantly associated with decreases in mental well-being over time. This suggests that the degree to which staff feel they are disrupted can impact their mental health – and that it is not necessarily correlated with actual exposure to patients with COVID-19 or levels of COVID-19 within the health board area. It is also notable that individual factors, such as gender, age or workload, did not have as great an effect on our cohort's mental health as reported in other studies,^7,10,15^ but that it was rather disruption resulting from COVID-19 itself that played a significant role in negatively affecting mental health. This is suggestive that systemic factors could have played a larger role in our cohort than individual factors, and has implications for policy, which often places emphasis on individual-level interventions.

### Mental well-being

Although this study did not identify independent factors protective of adverse mental health outcomes in our sample, we did observe mental well-being to be strongly negatively correlated with depression and anxiety at both times of measurement. Although mental well-being is seen as an umbrella concept incorporating various positive psychological constructs, it is its nurturing link to resilience that appears to be of importance for HSCWs during this pandemic. Recently, there have been calls to incorporate resilience training in medical education.^11^ In addition, there has been some evidence from this current pandemic that higher levels of personal resilience were associated with lower rates of negative mental health outcomes in HCWs.^15^ Although future studies would do well to identify possible protective factors, and the interplay between mental well-being and resilience, emphasis on enhancing personal mental well-being should not divert responsibility onto an individual's to simply ‘cope better’ with a challenging working environment.

### Proximity to COVID-19

Our study population were not working in a region with a high clinical burden of COVID-19 during the period under review. Healthcare workers in NHS Highland were nevertheless subject to lockdown measures, and most experienced disruption in their workplaces through service reconfiguration and infection control measures taken to help minimise transmission. Given the clinical need and common human impulse to access up-to-date information about the pandemic, it is likely that HSCWs in our study will have made use of COVID-19-related media, although this is not something we ascertained directly. Neill et al^34^ described increased anxiety and depression scores among individuals who accessed high levels of pandemic-related media coverage, and it is possible that this may have contributed to our findings.

### Limitations

Findings from the present study must be interpreted in light of its limitations. NHS Highland provides care for a population of 320 000 people over a wide geographical area, and employs around 10 000 staff.^35^ As such, the respondent sample represents approximately 2% of all staff employed by the local health board. Although 88% of respondents were female, this does not differ dramatically from the gender composition of the whole HSCW workforce in NHS Highland.^35^ The longitudinal aspect of the collected data was limited, and the identified trend toward worsening anxiety outcomes should be interpreted with caution. Although the maximum interval between measurements was 63 days, the follow-up period for most participants was approximately 40 days. Furthermore, those suffering from poor mental health may have been more likely to complete the surveys and thus potentially introduce self-selection bias into the findings. A further potential bias could be a result of attrition: participants who dropped out at follow-up, which could have affected the study's estimates. Participants were asked to self-report on their mental health, potentially introducing reporting bias. These potential biases, together with the small sample size and short follow-up period, places limitations on the generalisability of these findings. Additional longitudinal research that emphasises methodological rigor, including the use of standardised diagnostic interviews to establish mental health diagnoses, is necessary to better understand the mental health burden and identify those most at risk for adverse mental health outcomes in HSCWs.

In conclusion, our findings reveal that levels of anxiety and depression are a concern not only among HSCWs working in COVID-19 hotspots, but also for those in more remote settings like the Scottish Highlands. In contrast to what was observed in the general population, where studies found an improvement of mental health symptoms over time, our cohort's relatively high levels of anxiety and depressive symptoms persisted over time, raising concerns that this population may face immediate and ongoing adverse mental health consequences. Our findings suggest that although HSCWs with prolonged and high exposure to patients with COVID-19 need mental health support, it is also important not to overlook the negative mental health effects on all HSCWs. Mental health support is needed across different working contexts, and interventions to help staff cope with, understand and negotiate feelings of disruption may be beneficial.^13^ Although individual-level interventions that foster mental well-being and resilience may be beneficial, there is a need for wider, structural adaptations if we are to support the mental health of our HSCWs effectively. This could lead to resilient working systems, not just resilient individuals.^14,36^ Rigorous further longitudinal data are needed to respond to the potential long-term mental health effects of the COVID-19 pandemic on HSCWs.

## Data Availability

The data that support the findings of this study are available from the corresponding author, J.H.D.K., upon reasonable request.

## Supplementary material

For supplementary material accompanying this paper visit https://doi.org/10.1192/bjo.2021.1079. click here to view supplementary material
